# Involvement of cochlin binding to sulfated heparan sulfate/heparin in the pathophysiology of autosomal dominant late-onset hearing loss (DFNA9)

**DOI:** 10.1371/journal.pone.0268485

**Published:** 2022-07-28

**Authors:** Tomoko Honda, Norihito Kawasaki, Rei Yanagihara, Ryo Tamura, Karin Murakami, Tomomi Ichimiya, Naoki Matsumoto, Shoko Nishihara, Kazuo Yamamoto

**Affiliations:** 1 Department of Integrated Biosciences, Graduate School of Frontier Sciences, The University of Tokyo, Chiba, Japan; 2 Department of Bioinformatics, Graduate School of Engineering, Soka University, Hachioji, Tokyo, Japan; 3 Glycan & Life System Integration Center (GaLSIC), Soka University, Hachioji, Tokyo, Japan; University of Patras, GREECE

## Abstract

Late-onset non-syndromic autosomal dominant hearing loss 9 (DFNA9) is a hearing impairment caused by mutations in the coagulation factor C homology gene (*COCH*). *COCH* encodes for cochlin, a major component of the cochlear extracellular matrix. Though biochemical and genetic studies have characterized the properties of wild-type and mutated cochlins derived from DFNA9, little is known about the underlying pathogenic mechanism. In this study, we established a cochlin reporter cell, which allowed us to monitor the interaction of cochlin with its ligand(s) by means of a β-galactosidase assay. We found a class of highly sulfated glycosaminoglycans (GAGs), heparin, that were selectively bound to cochlin. The interaction was distinctly abrogated by *N*-desulfation, but not by 2-*O*- or 6-*O*-desulfation. The binding of cochlin to GAG was diminished by all of the point mutations found in DFNA9 patients. Through GAG composition analysis and immunostaining using mouse cochlin/immunoglobulin-Fc fusion protein, we identified moderately sulfated GAGs in mouse cochlea tissue; this implies that cochlin binds to such sulfated GAGs in the cochlea. Since GAGs play an important role in cell growth and survival as co-receptors of signal transduction mechanisms, the interaction of cochlin with GAGs in the extracellular matrix could aid the pathological research of autosomal dominant late-onset hearing loss in DFNA9.

## Introduction

The LCCL domain was identified as a conserved protein module in Limulus factor C, cochlear protein cochlin, and the late gestation lung protein Lgl1 [1]. Cochlin is a major component of cochlea protein [2] and consists of an N-terminal signal peptide and an LCCL domain, followed by two von Willebrand factor type A (vWA) domains [1]. The coagulation factor C homology gene *COCH* is responsible for autosomal dominant late-onset deafness disorder (DFNA9), which results in non-syndromic progressive sensorineural hearing loss associated with vestibular dysfunctions and usually appears from the second to fifth decade postpartum [3]. To date, several missense mutations and in-frame deletions in *COCH* have been identified in pedigrees afflicted with DFNA9 [4, 5]. The *COCH* gene product, cochlin, is a soluble protein specifically expressed in the cochlea [2, 3, 6, 7] where it is integrated into the extracellular matrix (ECM), according to immunohistochemical analysis [6, 7]. Recent proteomics studies have also revealed that cochlin is the most abundant protein in mammalian cochleae [2, 7, 8]. Morton’s group [9] developed Coch^-/-^ knock-out and Coch^G88E/G88E^ knock-in mice that exhibited an elevated auditory brainstem response at 13 and 21 months, respectively. Furthermore, Coch^-/+^ mice did not show hearing deficits, in contrast to Coch^G88E/+^ mice that showed elevated auditory brainstem response thresholds similar to those of Coch^G88E/G88E^ knock-in mice.

Biochemical and genetic studies have attempted to elucidate how mutations affect cochlin function. Correlations between the molecular properties of several mutated forms of cochlin and age of onset have been extensively studied in DFNA9 patients with regards to expression level [10], secreted amount (accumulated amount in the cell) [11], localization in the cell [11, 12], self-aggregation through dimer to multimer formation [5, 13, 14], cytotoxicity [14], deposition in the ECM [15], and endoplasmic reticulum-associated degradation via misfolding [16]. However, while these studies highlight the potential role of cochlin in regulating the survival of cochlear cells/tissue and ECM homeostasis, little is known about the specific molecular function of cochlin or its ligand(s) in the ECM.

The ECM consists of a panel of proteins, including several types of collagen and several species of glycosaminoglycans (GAGs) [17, 18]. The ultrastructural scaffold formed by ECM proteins promotes a variety of functions, including differentiation and maintenance of the cell, and tissue remodeling [19]. GAGs also play an important role in ECM homeostasis due to their hydrating properties and interaction with chemokines, growth factors, and other morphogens [17, 18].

The present study aimed at establishing a cochlin-expressing reporter cell [19] to identify cochlin ligand(s) within components of the ECM. We found that cochlin specifically bound to highly sulfated heparan sulfate (HS)/heparin (HP). All of the naturally occurring cochlin mutations found in DFNA9 patients caused a decrease in binding affinity between cochlin and GAGs. Furthermore, *N*-sulfation of GAGs was essential for cochlin binding. GAG containing both *N*- and *O*-sulfated disaccharide units was the major component in mouse inner ear tissue. Our findings demonstrate the role of cochlin in homeostasis of the cochlear ECM through their association with sulfated GAGs.

## Materials and methods

### Reagents and antibodies

Chondroitin (CH) from shark cartilage, chondroitin sulfate A (CS-A) from whale cartilage, chondroitin sulfate C (CS-C) from shark cartilage, dermatan sulfate (DS) from porcine skin, keratan sulfate from pocine nasal cartilage, and HS from pocine kidney were purchased from PG research (NaCS-A2, NACS-C2, NADS-B2, NSKS2, and NaHS-P2, Tokyo, Japan). HP from porcine intestinal mucosa was purchased from Calbiochem (375095, La Jolla, CA, USA). 2-*O*-desulfated heparin, 6-*O*-desulfated heparin, and *N*-desulfated, *N*-acetylated heparin were purchased from PG research (Tokyo, Japan). Heparinase I, heparinase II, and heparinase III from *Flavobacterium heparinum* were purchased from Sigma-Aldrich (St. Louis, MO, USA). Bovine serum albumin (BSA) and *N*-ethoxycarbonyl-2-ethoxy-1,2-dihydroquinoline (EEDQ) were purchased from Wako Pure Chemicals (Tokyo, Japan). Hyaluronic acid (HA) from *Streptococcus zooepidemicus* was provided by Shiseido Co. (Tokyo, Japan). Anti-human and mouse cochlin polyclonal antibody (Ab) was purchased from Lifespan Biosciences (LS-C334796, Seattle, WA, USA).

### Cell lines

BWZ.36 cells were provided by Dr. N. Shastri (University of California Berkeley, Berkeley, CA, USA) and maintained in RPMI 1640 medium (Sigma-Aldrich) supplemented with 10% heat-inactivated fetal calf serum (FCS, Gibco, Tokyo, Japan), 100 U/mL penicillin, 100 μg/mL streptomycin, 2 mM glutamine, and 50 μM 2-mercaptoethanol under 5% CO_2_ at 37°C. The retrovirus-packaging cell line, Plat-E cells, were provided by Dr. T. Kitamura (The University of Tokyo, Tokyo, Japan) and maintained in Dulbecco’s modified Eagle’s medium (Sigma-Aldrich) supplemented with 10% heat-inactivated FCS, 100 U/mL penicillin, 100 μg/mL streptomycin, 2 mM glutamine, 50 μM 2-mercaptoethanol, and 20 mM HEPES (D10 medium). HEK293 cells (Riken cell bank, Ibaraki, Japan) were maintained in D10 medium. Experiments using human cell lines and genetic recombination experiments were conducted in accordance with a comprehensive, high quality care program, which was approved by the Life Science Research Committee of the Graduate School of Frontier Sciences at The University of Tokyo and the Life Science Committee of The University of Tokyo.

## Cochlin cDNA

A cDNA encoding human cochlin was obtained from FLJ cDNA clone FLJ41368 (Toyobo, Tokyo, Japan). Because the FLJ41368 clone had an inadequate insertion compared to that of human cochlin cDNA (Genbank: NM_004086.1), we removed the insertion by polymerase chain reaction (PCR) using the FLJ41368 cDNA clone as template and cloned the amplified human cochlin cDNA into pBluescript II SK(+) (Stratagene, La Jolla, CA). DNA sequencing of the recloned cochlin cDNA showed two point mutations: C1055G is a missense mutation that causes an amino acid substitution from threonine to serine and is reported as a single nucleotide polymorphism (SNP, Japanese SNP database [http://snp.ims.u-tokyo.ac.jp/index.html], ID: IMS-JST004957); C1356T is a silent mutation and is not reported as a SNP. The cDNA encoding mouse cochlin (Genbank: NM_00729.5) was amplified by PCR from mouse spleen cDNA, using primers mcochlin-F and mcochlin-R (S1 Table). The amplified product was subcloned into the pBluescript II SK(+) plasmid.

### Construction of plasmids for wild-type and mutated cochlin chimeric proteins

The pMXs-IRES-EGFP retroviral vector (pMXs-IG, a gift from Dr. T. Kitamura, The University of Tokyo, Tokyo, Japan) was modified to include a CD3ζ cassette encoding the intracellular region of CD3ζ at the 3’ terminus of the cloned gene [19, 20]. pMXs-IG contains an internal ribosome entry site followed by the enhanced green fluorescent protein (EGFP) gene, which permits simultaneous expression of a cloned gene and EGFP. The CD3ζ cassette was generated by PCR using primers CD3ζ-F and -R (S1 Table) and pBlue-CD3ζ as a template. The PCR product was cloned into pBlueScript II SK(+) and its sequence was verified by sequencing. The CD3ζ cDNA digested with XhoI and NotI was cloned into pMXs-IG digested with the same enzymes. The plasmid, pMXs-IG-CD3ζ, was used as a vector for the expression of wild-type and mutant cochlin-CD8α-CD3ζ chimeric proteins. The CD8α-CD3ζ chimeric protein, cloned in the same vector, was used as a negative control in the GAG-binding assay.

**Fig 1:**
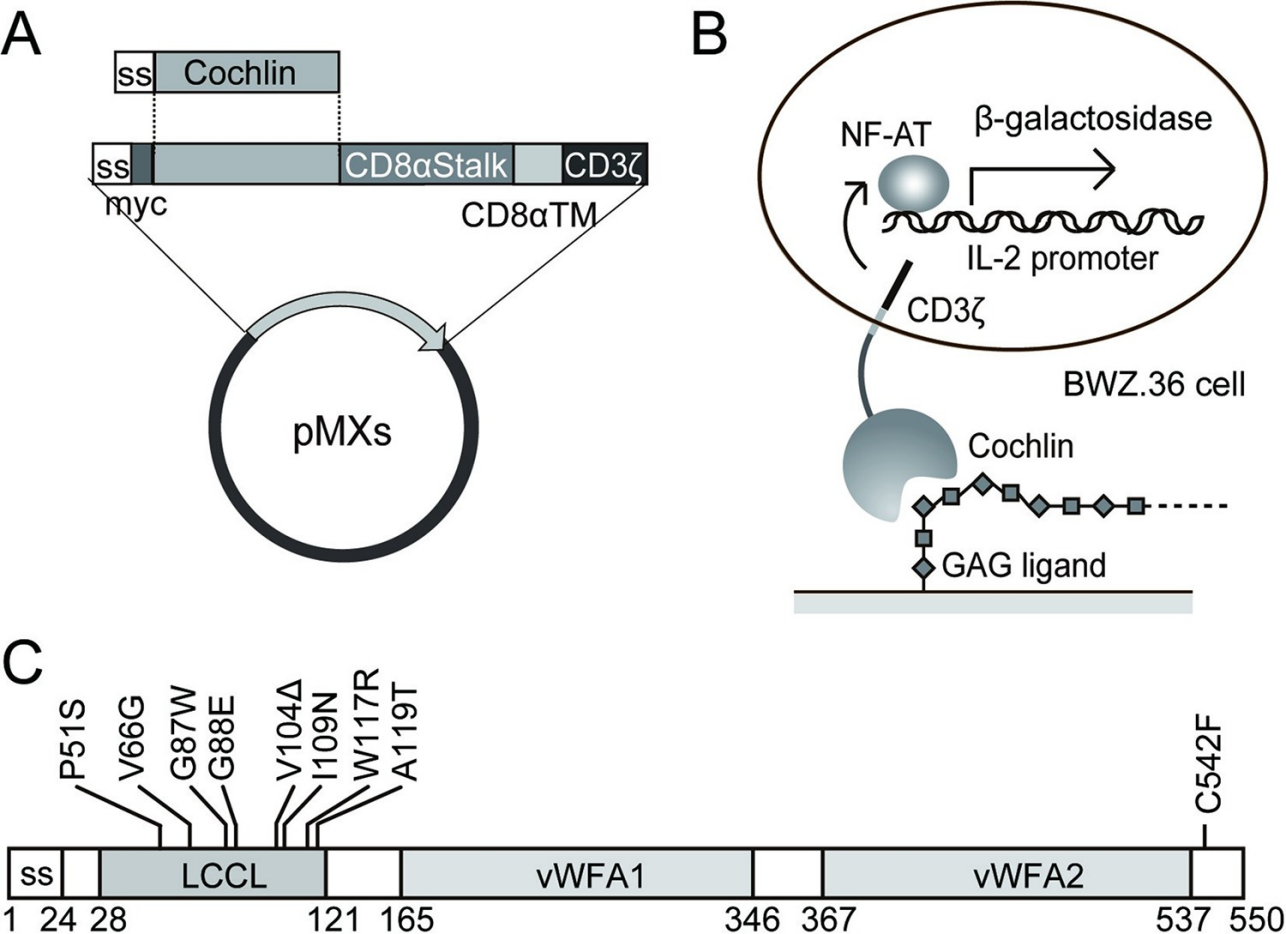
Establishment of cochlin reporter cells and domain organization of cochlin showing point mutations of DFNA9 patients. (A) Expression plasmid for the expression of chimeric cochlin/CD8α/CD3ζ protein. (B) Schematic illustration of the cochlin reporter cell, cochlin-BWZ. Cochlin is anchored on the cell surface by fusion with the stalk and transmembrane regions of CD8α followed by the intracellular domain of CD3ζ. Crosslinking of cochlin with its ligands transduces the signal via CD3ζ NA-AT to enhance β-galactosidase expression. (C) Domain structure of human cochlin and several point mutations found in DFNA9 patients [4, 16, 27, 28].

Human cochlin-CD8α cDNA was generated by overlap extension PCR. Briefly, the cDNA encoding human cochlin and the cDNAs encoding the stalk and transmembrane regions of CD8α were PCR-amplified using primers Cochrep-F and -R (S1 Table) with a pBlue-cochlin template. Using these PCR products as templates, the cDNA encoding cochlin-CD8α was amplified by overlap extension PCR using primers Cochrep-F and CD8α-R and cloned into pBlueScript II SK(+). The cochlin-CD8α cDNA digested with XhoI and ScaI was cloned between the XhoI and HpaI site of pMXs-IG-CD3ζ to obtain pMXs-hCoch-CD8α-CD3ζ (Fig 1A). Mutations of the human cochlin cDNAs found in DFNA9 patients (P51S, V66G, G87W, G88E, V104Δ, I109N, W117R, A119T, and C542F) were introduced with the QuickChange site-directed mutagenesis kit (Stratagene) according to the manufacturer’s protocol and using the primers listed in S1 Table. To detect the expression of the cochlin-CD8α-CD3ζ fusion protein on the cell surface, we added a Myc-tag sequence upstream of the cochlin-CD8α-CD3ζ cDNA. Similarly, we constructed pMXs-mCoch-CD8α-CD3ζ to express a mouse cochlin-CD8α-CD3ζ fusion protein on the cell surface. The final constructs obtained were pMXs-myc-hCoch-CD8α-CD3ζ and pMXs-myc-mCoch-CD8α-CD3ζ (Fig 1A).

### Establishment of BWZ.36 cell lines expressing wild-type and mutant cochlin

Retroviral transduction was performed according to the method of Toshio Kitamura et al. [20]. Plat-E cells were transfected with pMXs-hCoch-CD8α-CD3ζ, pMXs-hCoch(P51S)-CD8α-CD3ζ, pMXs-hCoch(V66G)-CD8α-CD3ζ, pMXs-hCoch(G87W)-CD8α-CD3ζ, pMXs-hCoch(G88E)-CD8α-CD3ζ, pMXs-hCoch(V104Δ)-CD8α-CD3ζ, pMXs-hCoch(I109N)-CD8α-CD3ζ, pMXs-hCoch(W117R)-CD8α-CD3ζ, pMXs-hCoch(A119T)-CD8α-CD3ζ, or pMXs-hCoch(C542F)-CD8α-CD3ζ, using Lipofectamine 2000 (Invitrogen, San Diego, CA, USA) according to the manufacturer’s protocol. The pMXs-myc-CD8α-CD3ζ plasmid was used as a negative control for cochlin expression. Two days after transfection, each culture supernatant was isolated and added to the BWZ.36 cell culture, with 8 μg/mL polybrene. A BWZ.36 cell line expressing mouse cochlin on the cell surface was established using pMXs-mCoch-CD8α-CD3ζ.

### Preparation of GAG-coupled BSA

Coupling of GAGs to BSA using EEDQ was performed according to the method of Ishitsuka R. et al. [21]. Briefly, 5.0 mg of GAG dissolved in 400 μL of distilled water were added to 500 μL of 10 mg/mL EEDQ in ethanol and incubated for 1 h at 25°C. After the reaction mixture was cooled at 4°C, 500 μL of 20 mg/mL BSA in distilled water was added and incubated for 12 h at 4°C. The reaction solution was concentrated to 500 μL in TBS (20 mM Tris-HCl [pH 8.0], and 150 mM NaCl) by ultrafiltration using an Amicon Ultra-15 (Amicon, Beverly, MA, USA). For chondroitin and hyaluronic acid, 1.0 mg of GAG and 2.0 mg of BSA dissolved in 450 μL of distilled water were added to 500 μL of 5.0 mg/mL EEDQ in ethanol and incubated for 12 h at 4°C. The concentration of GAG-BSA was assessed with a BCA protein assay kit (Pierce, Rockford, IL, USA).

### Reporter assay

A 96-well enzyme-linked immunosorbent assay (ELISA) plate was coated with 30 μL/mL of GAG-BSA, 5 μg/mL antibodies, collagen, fibulin, or laminin in 10 mM phosphate buffer (pH 7.4) containing 150 mM NaCl (PBS). The plate was washed with PBS and each well was seeded with 1.0 × 10^5^ human cochlin-expressing BWZ.36 (hCoch-BWZ) cells and mutant cochlin-expressing BWZ.36 (hCoch(P51S)-BWZ) cells, and cultured for 16 h. After incubation, cells were washed with PBS, lysed with PBS containing 0.125% NP-40, 9.0 mM MgCl_2_, 100 mM β-mercaptoethanol, and 0.15 mM chlorophenol red-β-galactopyranoside (CPRG, Wako Pure Chemicals) and incubated for 2–4 h. β-galactosidase activity was measured as absorbance at 570 nm with 630 nm as the reference wavelength. The final plot included values of absorbance at 570 nm from which the absorbance at 630 nm had been subtracted.

### Expression and purification of a mouse cochlin-Fc fusion protein and immunostaining using cochlin-Fc

To express mouse cochlin fused to human IgG-Fc (mcochlin-Fc), mouse cochlin cDNAs were amplified by PCR and inserted between the EcoRI and XhoI sites of the pCAGGS-Fc vector [22]. HEK293 cells were transfected with purified plasmid using Lipofectamine 2000 and cultured for a week in D10 medium containing 2 μg/mL puromycin. mcochlin-Fc was purified from the cultured media using a column of protein A-Sepharose (GE healthcare, Tokyo, Japan). To evaluate the expression of mcochlin-Fc, purified proteins were separated by SDS-polyacrylamide gel under reducing conditions, and then transferred to a PVDF membrane. After blocking with 0.5% skim milk in TBS containing 0.1% Tween-20 (TBS-T), the membrane was incubated with mouse anti-Myc antibody (9E10, Santa Cruz Biotechnology, Dallas, TX, USA) at a dilution of 1:500 followed by staining with horseradish peroxidase (HRP)-conjugated anti-mouse IgG (Jackson Immunoresearch Laboratories, West Grove, PA, USA) at a dilution of 1:1,000. After washing with TBS-T, the membrane was incubated with Immobilon Western chemiluminescent HRP substrate (Millipore, Billerica, MA, USA). The protein content was estimated by measuring the density of the CBB-stained band and extrapolating it to a calibration curve with a known concentration of BSA.

### Enzyme-linked immunosorbent assay (ELISA)

A 96-well ELISA plate was coated with 15 μg/mL of GAG-BSA for 18 h at 4°C, and then blocked with TBS containing 3% skim milk for 1 h at room temperature. mcochlin-Fc, diluted with TBS, was added to the wells and incubated for 1 h at 37°C. After washing with TBS-T three times, wells were incubated with 1:5,000 diluted HRP-conjugated anti-human IgG for 1 h at 37°C. After washing with TBS-T five times, wells were incubated with Sigmafast OPD (Sigma-Aldrich) for 10–30 min at room temperature. Absorbance was measured at 450 nm.

### Extraction of GAGs from mouse inner ear tissue and analysis of the disaccharide composition of heparan sulfate

Mouse tissue was obtained in accordance with a comprehensive, high quality care program, approved by the Life Science Research Committee of the Graduate School of Frontier Sciences at The University of Tokyo and the Life Science Committee of The University of Tokyo. The inner ear tissues of 8-week-old female C57BL/6 mice were dehydrated and delipidated by homogenization in ice-cold acetone. Extraction of GAGs was performed as previously described [23]. The recovery of GAGs was calorimetrically determined at 540 nm by a carbazole reaction. The disaccharide composition of HS/HP was determined by ion-exchange chromatography. In brief, the prepared GAG fraction was incubated with heparinase I, II, and III in 0.1 M NaOAc (pH 7.0), containing 3.7 mM CaCl_2_, at 37°C for 18 h. The reaction mixture was then derivatized with 2-aminobenzoic acid (2AB), and recovered by gel chromatography on a column of Superdex peptide (3 × 250 mm, GE Healthcare). Disaccharide fractions were separated on a column of Hitrap Q (7 × 25 mm, GE Healthcare) or Hitrap DEAE fast flow (7 × 25 mm, GE Healthcare). Identification and quantification of the disaccharides was performed by comparison with standards, which were prepared from heparinase digestions of heparin, 2-*O*-desulfated heparin, 6-*O*-desulfated heparin, *N*-desulfated heparin, and heparan sulfate.

### Immunohistochemistry of mouse inner ear sections

The 8-week-old C57BL/6 mice (Japan SLC, Shizuoka, Japan) were euthanized with diethyl ether and inner ear tissues were isolated and fixed using 4% paraformaldehyde dissolved in PBS at 4°C for 18 h. Inner ear tissues were then placed in 120 mM EDTA for 1 week, which was successively switched to 70%, 80%, and 99.5% ethanol. Dehydrated inner ear tissues were immersed in xylene followed by a 1:1 mixed solution of xylene and paraffin at 65°C. The inner ears were embedded in paraffin, sliced to a thickness of 15 μm using a microtome, attached to a glass slide (MAS-GP type A, Matsunami Glass, Osaka, Japan), and successively immersed in xylene, 99.5% ethanol, 80% ethanol, 70% ethanol, and distilled water. For hematoxylin and eosin (H&E) staining, deparaffinized sections were immersed in Mayers hematoxylin solution (Wako Pure Chemicals) and allowed to stand at room temperature for 5 min. After washing with tap water, counterstaining was performed in 0.1% eosin Y in ethanol (Wako Pure Chemicals) and allowed to stand at room temperature for 5 min. Digestion of HS was performed with heparinase I, II, and III (Sigma-Aldrich, 16 mU/mL) in 50 mM acetate buffer (pH 7.0), containing 25 mM CaCl_2_, at 37°C for 2 h. For staining with the mouse cochlin-Fc fusion protein, deparaffinized sections were treated with LAB solution (Polysciences, Warrington, PA, USA) to activate antigens according to the manufacturer’s protocol. After washing twice with 10 mM Tris-HCl (pH 8.0) and 150 mM NaCl (TBS), blocking was performed with 10% goat serum (Sigma-Aldrich) at room temperature for 1 h. After washing twice with TBS, the cochlin-Fc fusion protein (10 μg/mL) was added to the sample sections at 4°C for 18 h. After washing three times with TBS, an alkaline phosphatase-labeled anti-human IgG Fc antibody was added to the sample sections and allowed to stand at room temperature for 1 h. After washing five times with TBS, the sections were placed in INT/BCIP (165 μg/mL, Sigma-Aldrich) suspended in 100 mM Tris-HCl, 100 mM NaCl, 50 mM MgCl_2_, and 0.1% Triton-X100 at 37°C for 20–60 min. Experiments using animals were conducted in accordance with the guidelines for proper conduct of animal experiments and was approved by the Life Science Research Committee of the Graduate School of Frontier Sciences at The University of Tokyo and the Life Science Committee of The University of Tokyo (permit number: C-15-04).

### Statistical analyses

The data of reporter assay and ELISA were expressed as means ±SE from three or more experiments. An unpaired Student’s t-test was used to compare the growth or intensity of the bands in the two experimental groups.

## Results

**Fig 2:**
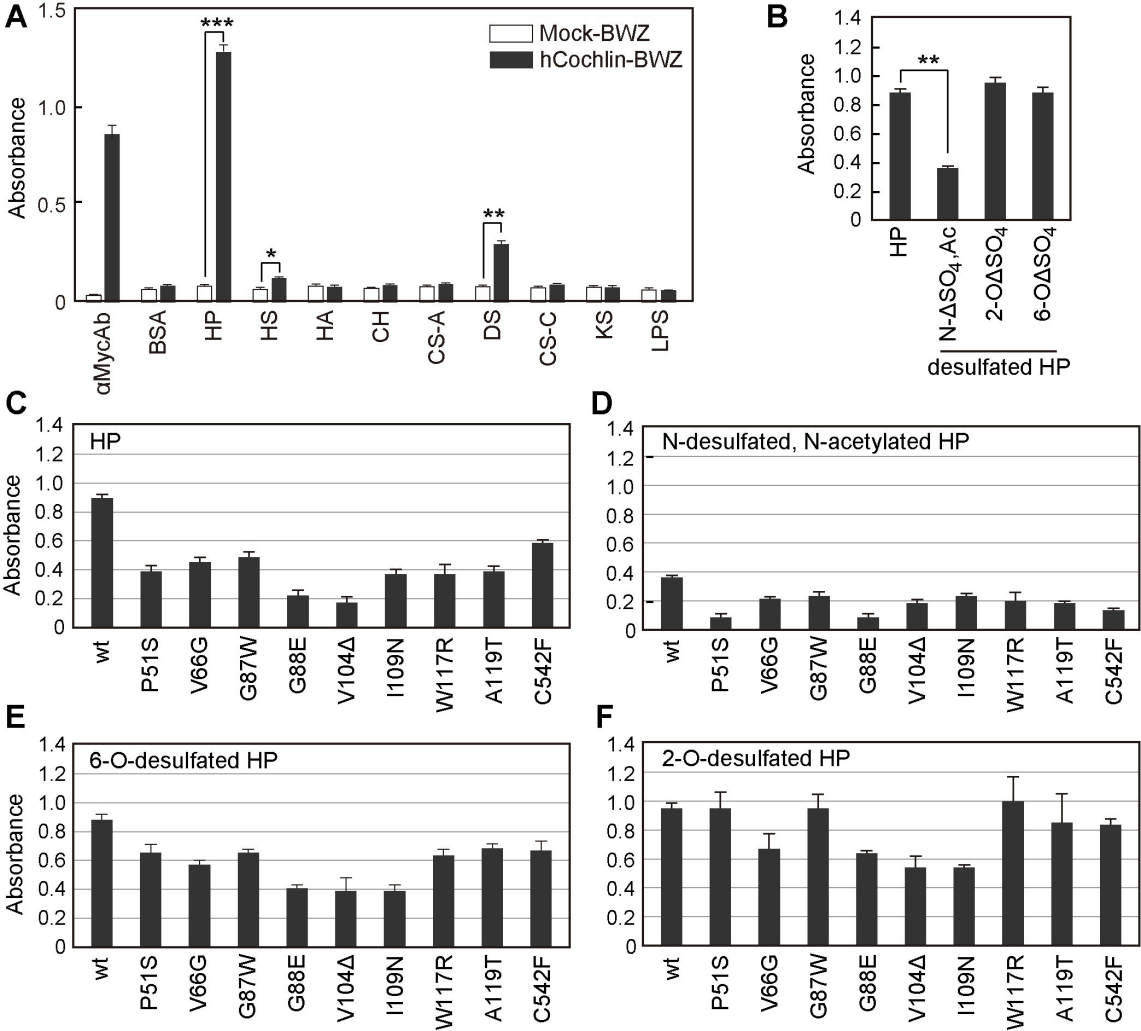
Human cochlin bound to *N*-sulfated heparin and mutations in DFNA9 patients decreased binding affinity. (A) Reporter assay of the interaction between human cochlin and several extracellular matrices. (B) *N*-desulfated, *N*-acetylated heparin significantly decreased binding affinity for cochlin. Each wild-type and mutated cochlin reporter cell was cultured in a well coated with (C) heparin, (D) *N*-desulfated, *N*-acetylated heparin, (E) 6-*O*-desulfated heparin, or (F) 2-*O*-desulfated heparin for 18 h at 37°C and β-galactosidase expression was monitored calorimetrically. The data are represented as the mean ± SD of n = 3 independent wells. *** *p* < 0.005; ** *p* < 0.01; * *p* < 0.05 (Student’s *t*-test).

Myc-tagged human cochlin, fused to the CD8α stalk and transmembrane domains followed by the CD3ζ cytoplasmic domain, was expressed on the surface of BWZ.36 cells (hcochlin-BWZ), which contain a β-galactosidase reporter gene under the control of an IL-2 promoter (Fig 1B). Crosslinking of cochlin on the surface of the cells causes β-galactosidase expression (Fig 2A). To identify cochlin ligand(s) and especially glycosaminoglycans (GAG) in the inner ear ECM, each GAG was immobilized and hcochlin-BWZ cells were cultured in the GAG-coated wells. Expression of the cochlin fusion protein was confirmed by β-galactosidase induction by culturing the cells in anti-Myc Ab-immobilized wells (Fig 2A). When heparin (HP)-BSA was immobilized, hcochlin-BWZ distinctly produced β-galactosidase (Fig 2A), indicating that human cochlin was preferentially bound to HP. DS showed weak interaction with human cochlin; however, the other GAGs (Fig 2A), and matrix proreins such as collagen type I, II, III, IV, and V, and fibronectin, did not induce β-galactosidase production in hcochlin-BWZ cells (S1 Fig). HP is a highly sulfated GAG; thus, we examined the effect of the HP sulfation position on cochlin binding. *N*-desulfated, *N*-acetylated HP exhibited a significant reduction in cochlin binding affinity, whereas no effect on binding was observed for either 2-*O*-desulfated or 6-*O*-desulfated HP (Fig 2B), indicating a specificity of human cochlin for binding to *N*-sulfated HP. Next, we prepared several mutated cochlin-BWZ cells with mutations naturally occurring in DFNA9 patients (Fig 1C) and examined their interaction with HP and desulfated HPs. The cochlin mutants such as P51S, G87W, and W117R had lower binding to heparin, *N*-desulfated heparin, and 6-*O*-desulfated heparin than wild-type, but their binding to 2-*O*-desulfated heparin remains similar to wild-type (Fig 2C–2F). In contrast, the cochlin mutants, G88E, V104Δ, and I104N, had weak binding affinity to 2-*O*-desulfated heparin (Fig 2F), suggesting the possibility of some selective interactions of these mutated residues with different types of sulfate groups. Because most of the point mutations from DFNA9 patients are concentrated to the LCCL domain, our findings suggest that cochlin may bind to sulfated heparan sulfate via its LCCL domain, and that this interaction is weaker when several point mutations are introduced in the *COCH* gene.

**Fig 3:**
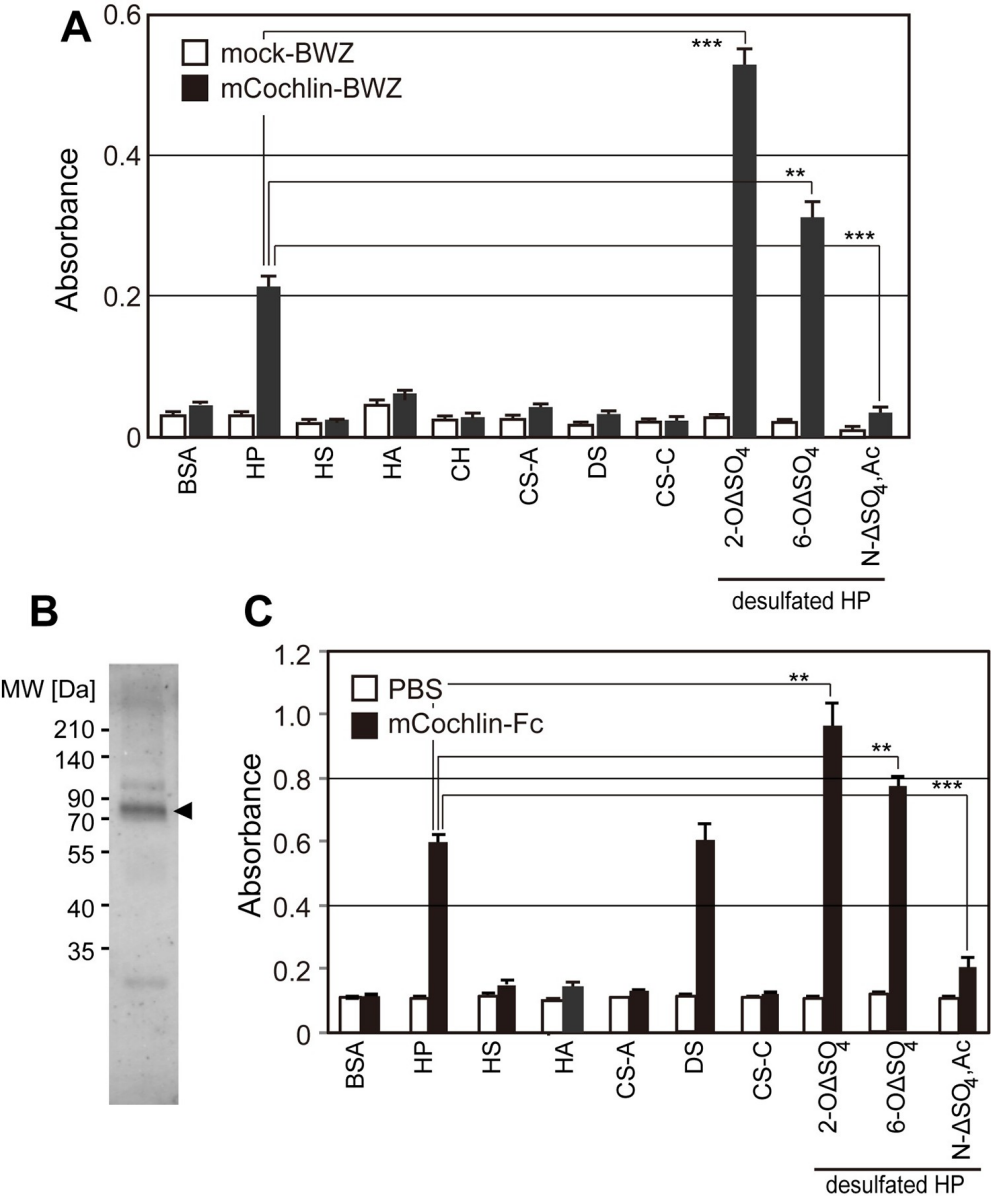
Binding of mouse cochlin to heparin and its desulfated derivatives. (A) Reporter assay of the interaction between mouse cochlin and several glycosaminoglycans. (B) Sodium dodecyl sulfate-polyacrylamide gel electrophoresis (SDS-PAGE) of purified mouse cochlin-Fc fusion protein (mcochlin-Fc). (C) Binding of mcochlin-Fc to immobilized GAG-BSA was analyzed by ELISA. *** *p* < 0.005; ** *p* < 0.01 (Student’s t-test).

We examined the interaction of mouse cochlin with several GAGs using established mcochlin-BWZ cells. Mouse cochlin also bound to HP and *N*-desulfation/*N*-acetylation of HP led to a significant reduction in binding affinity, similar to that observed with human cochlin (Fig 3A). However, mouse cochlin binding to HP was enhanced by 2-*O*- or 6-*O*-desulfation. To further investigate the preference of mouse cochlin-binding to HP and desulfated HPs, ELISA was performed by preparing mouse cochlin fused to human IgG-Fc (mcochlin-Fc) as previously described [22] (Fig 3B). The binding data of mcochlin-Fc to immobilized GAGs were similar to the data obtained by mouse cochlin-expressing reporter assay (Fig 3C), and mcochlin-Fc binding to 2-*O*-desulfated, and 6-*O*-desulfated heparin were increased compared to that to heparin. In ELISA assay, mcochlin-Fc obviously bound to dermatan sulfate (DS) although mouse cochlin reporter cells did not react with DS. To investigate whether HP or highly sulfated HS is present in mouse cochlea tissue, a GAG fraction was prepared from the inner ear tissue of C57BL/6 mice and the composition of disaccharides derived from HS/HP was examined as previously described [23]. Trisulfated disaccharide was not detected and disaccharides derived from 2-*O*- or 6-*O*-desulfated, *N*-sulfated HS were dominantly detected (31.7%, Table 1).

**Table 1:** GAG composition of cochlear tissue from 8-week-old female C57BL/6 mice.

disaccharide units	nonsulfated	monosulfated	disulfated		trisulfated
			2-*O*-, *N*-di	6-*O*-, *N*-di	2-*O*-, 6-*O*-di
relative content (%)	5.8	62.5	31.7	ND	ND

GAG fraction prepared from cochlear tissues (three 8-week-old female mice) was analyzed. 2-*O*-, *N*-disulfated and 6-*O*-, *N*-disulfated disaccharides were eluted together from Hitrap Q or Hitrap DEAE fast flow columns. ND; not detected. Representative chromatogram was shown in S2 Fig.

**Fig 4:**
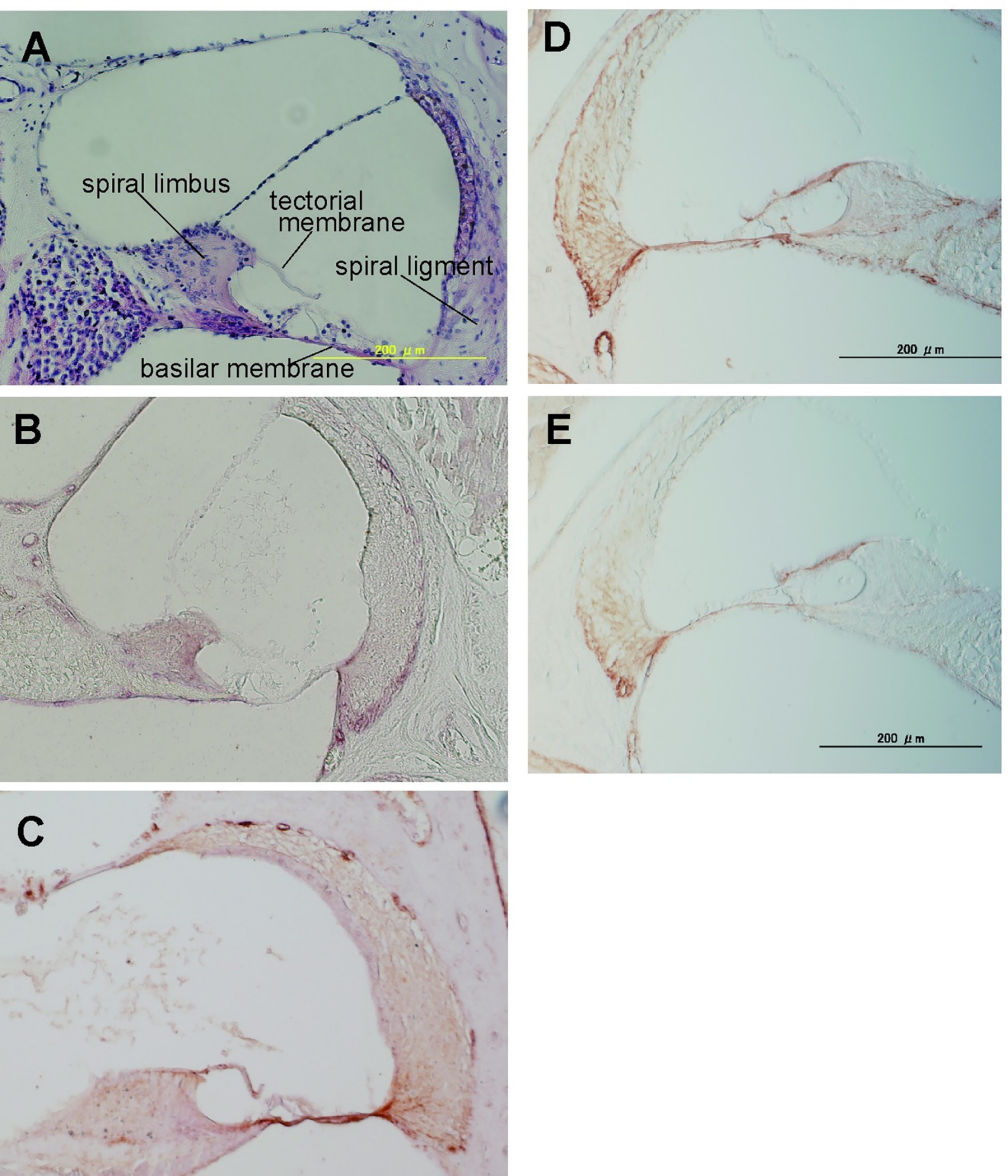
Histochemical staining of mouse inner cochlear tissue using mcochlin-Fc. Paraffin-embedded serial sections of C57BL/6 female mouse inner cochlear tissue was stained with (A) H&E, (B) anti-cochlin Ab, or (C) mcochlin-Fc. The same serial sections were stained with mcochlin-Fc without (D) or with heparinase treatment (E). Scale bar = 200 μm.

To evaluate the presence of the GAG ligand in mouse cochlea tissue, mouse cochlin fused to human IgG-Fc (mcochlin-Fc) was prepared as previously described [22] (Fig 3B). In the ELISA, mouse cochlin-Fc was observed binding only to HP in the tested GAGs (Fig 3C), indicating that both human and mouse cochlin bind specifically to HP. Then, by using mcochlin-Fc and anti-cochlin antibody as probes, histochemical staining of paraffin-embedded mouse cochlea tissue sections was performed. H&E staining clearly visualized the main structure of the cochlea (Fig 4A). Cochlin was localized to the basilar membrane, spiral limbus, and marginal zone of the spiral ligament (Fig 4B). Staining of the cochlin ligand with mcochlin-Fc showed similar distribution patterns, in addition to the tectorial membrane (Fig 4C). Our findings indicated that cochlin and its ligands were co-localized in the basilar membrane, spiral limbus, and marginal zone of the spiral ligament. To confirm whether the cochlin ligands were highly sulfated glycosaminoglycans, staining of mcochlin-Fc was performed after pre-treatment with heparinase I, II, and III. The efficacy of staining with mcochlin-Fc was reduced by heparinase treatment (Fig 4D and 4E), indicating that sulfated HS was the major ligand of cochlin in the inner cochlear tissue. However, the staining signal with mcochlin-Fc toward inner cochlear tissue sections was clearly detected even after heparinase pre-treatment indicating that DS may be present in inner cochlear tissue and was detected with mcochlin-Fc because mcochlin-Fc could bind to DS also (Fig 3C).

## Discussion

Familial cases of autosomal dominant sensorineural hearing loss have been documented over the last 90 years [24–26]. The genetic and audiologic manifestations differ in the initial age at onset of hearing loss, rates of disease progression, and in the occurrence of tinnitus. Many studies have focused on understanding the characteristic pathology of DFNA9 based on the biochemical, immunochemical, and genetic characteristics of cochlin itself [4, 16, 27, 28]. However, little is known about the function of cochlin or its ligand(s). The present study revealed that cochlin binds to a component of the ECM, GAG, especially to sulfated HS. Heparin consisting of trisulfated disaccharide did not exist but moderately sulfated HS consisting of 2-*O*- and *N*-disulfated disaccharide and/or 6-*O*- and *N*-disulfated disaccharide was dominantly expressed in the inner ear, suggesting that cochlin could bind to these GAGs. In the binding experiments using cochlin-expressing reporter cell and mouse cochlin-Fc, binding to porcine HS was not detected (Figs 2 and 3). Presumably the porcine HS used in this experiment had low sulfation, which was suported by the previous report showing that disaccharide structures of porcine HS was tri-S 4.1%, di-S 11.9% (2S+NS 8.6%, NS + 6S 3.3%), and mono-S 30.2%, respectively [29].

This study demonstrated that the expression level of the cochlin ligand is potentially more important in determining the characteristic pathology of DFNA9 than cochlin itself or the interaction between cochlin and the cochlin ligand. Interestingly, all of the mutated cochlins from DFNA9 patients [4], P51S, V66G, G87W, G88E, V104Δ, I109N, W117R, and A119T, exhibited a reduction in their GAG-binding ability, though to varying degrees (Fig 2). Such point mutations of cochlin did not affect their stability and expression level in reporter cells when FACS analysis was performed with an anti-Myc antibody. Furthermore there was not so much difference in the yield when each mutated cochlin-Fc fusion protein was prepared. GAG-​​binding specificities of mouse and human cochlins showed the same tendency for decreased binding to *N*-desulfated heparin compared with the binding to heparin, however binding propeties of these proteins against 2-*O*-deulfate and 6-*O*-desulfated heparin were apparantly different. Mouse and human cochlins have approximately 95% homology at the amino acid level, and the LCCL domains of these two proteins also show 94% homology each other. Comparing between mouse and human cochlins, five amino acid residues have significantly different properties (A31P, P51S, Y62F, S84G, T122A). Thus these amino acid residues may contribute to the different binding preference to the partially desulfated heparin. Since these point mutations are located in the LCCL domain, the results also indicated that cochlin may interact with GAGs via this domain. A lipopolysaccharide-activated serine-protease, Limulus factor C, participates in coagulation of horseshoe carb hemolymph and the LCCL domain of Limulus factor C is involved in LPS binding [30]. Thus, the LCCL domain may be a lectin-like domain that can bind to pathogen-associated molecular patterns as well as GAGs. HS/HP, the major constituent of the ECM, contains repeating disaccharide units consisting of amino sugar and uronic acid, which frequently show heterogeneity in degree of sulfation, sulfation position, and epimerization. GAGs are well-recognized components of the extracellular microenvironment, and function as co-receptors of various GAG-binding molecules, including growth factors and chemokines; thus, GAG structures could be related to the signals for proliferation, differentiation, and survival of the cells. Interestingly, both sulfation level and sulfation position in HS/HP are gradually altered in an age-dependent manner [31, 32]. Gradual structural changes in GAGs could explain early onset deafness in DFNA9 patients with various point mutations in the *COCH* gene. During the preparation of GAG fraction from inner ear tissues of young and old mice, we observed that amount of glycosaminoglycans derived from inner cochlear tissues was decreased in old mice (13-month-old) compared with young mice (8-week-old) (S3A Fig). In another experiment, we measured the amount of mRNAs encoding HS-2-*O*-sulfotransferase (*hs2st*), HS-6-*O*-sulfotransferase 1 (*hs6st1*), HS-6-*O*-sulfotransferase 2 (*hs6st2*) and *N*-sulfotransferase (*ndst*) by using real-time PCR experiment. Though *hs2st* cDNA was not successfully amplified, the results showed that expression level of *hs6st1* was slightly decrased in old mice but mRNAs encoding other sulfotransferases were not altered between young and old mice (S3B Fig).

**Fig 5:**
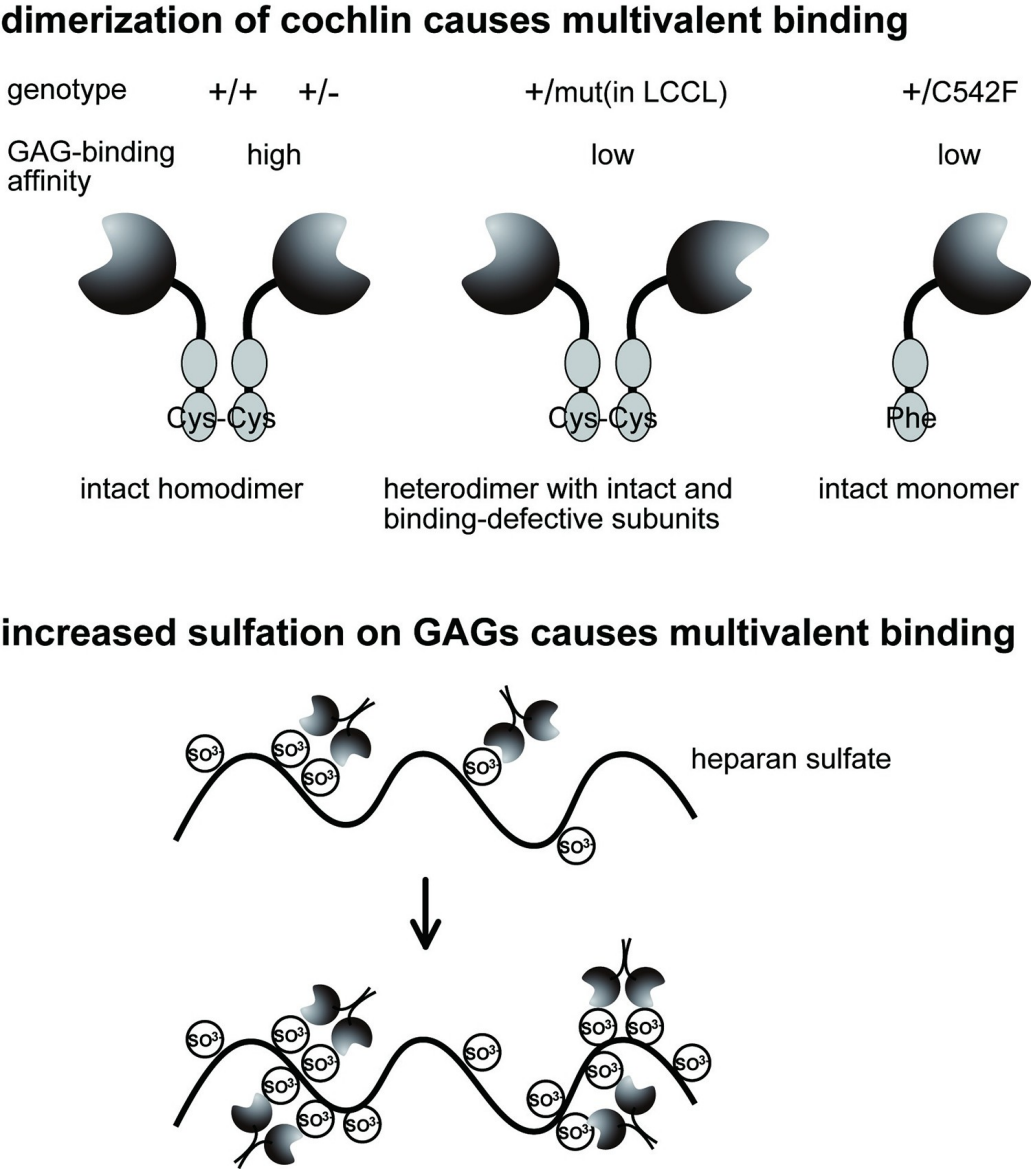
The effects of cochlin dimerization and GAG sulfation on their binding. GAG binding is maximized in wild-type cochlin homodimers (left). Dimers formed between wild-type cochlin and mutated cochlin (middle) as well as a cysteine substitution in cochlin (C542F) that inhibits dimer formation via a disulfide bridge (right) reduced cochlin’s GAG-binding ability. Given the age-dependent decrease of heparan sulfate/heparin contents and their sulfation levels, the interaction between cochlins and GAGs provide a possible mechanism for understanding autosomal dominant, late-onset hearing loss in DFNA9. Sulfation of GAGs is also involved in the interaction between cochlin and sulfated GAG because increased sulfation of GAGs presumably causes increased binding sites of cochlin.

Lectin and sugar interactions are weaker than protein–protein interactions, with typical *Ka* values of around 10^4^ M^-1^. Thus, lectins usually form dimers or tetramers, with oligomerization greatly enhancing their sugar-binding ability. Mono-, di-, tri-, and tetravalent *Griffonia simplicifolia* lectins bind to human type A erythrocyte with *Ka* values of 7.5 × 10^5^, 2.9 × 10^6^, 1.4 × 10^7^, and 1.2 × 10^7^ M^-1^, respectively [33]. In nature, cochlins can form dimers [14], which may be essential for binding to extracellular GAGs. When only a wild-type cochlin was expressed, cochlin formed an intact dimer with a high affinity (Fig 5). However, when wild-type and mutated cochlins were expressed at equal levels in heterozygous carriers of the mutated cochlin allele, cochlin predominantly formed heterodimers consisting of wild-type and mutated protein forms. These heterodimers are predicted to show a lower affinity than wild-type homodimers since one of the two binding sites on the dimer are defective for GAG binding. This hypothesis clearly explains why *COCH* mutations are dominantly inherited in DFNA9 kindreds and the phenotypes of Coch^-/-^, Coch^-/+^, Coch^G88E/G88E^, and Coch^G88E/+^ mice [9] corroborate this hypothesis. In DFNA9 patients, mutations are mainly located in the gene encoding the LCCL domain of cochlin. However, other mutants without a mutation in the LCCL domain have also been reported and mostly involve Cys residues (C162Y, C542Y, C542F) [4, 34]. Although the quaternary structure of cochlin mutants has not yet been determined, Cys residues may participate in the formation of stable dimers through inter-chain disulfide bond formation. Substitution of these Cys residues with Tyr or Phe may abrogate stable homo- and heterodimer formation (Fig 5), resulting in lower affinity of Cys-mutated cochlin to highly sulfated HS or HP (C542F, Fig 2C). In the case of GAGs as ligands, multiple binding sites may exist on a single GAG chain. If sulfations of GAGs are involved in the binding of cochlin, increased sulfation of GAGs may cause increased number of cochlin binding sites. Because multiple binding is important for the strong interaction between sugar and lectin interactions, both dimerization of cochlin and GAG sulfatiom may be important for biological activity and maintenance.

In summary, this is the first report of human and mouse cochlin binding to heparin and moderately sulfated HS. Most of the mutated cochlins found in DFNA9 patients displayed lower GAG-binding ability than wild-type cochlin. Given the known age-dependent decreases in HS/HP content and sulfation level, our findings concerning cochlin ligands provide novel insights into the mechanism underlying the clinical phenotype of autosomal dominant, late-onset hearing loss.

## Supporting information

S1 Fig Human cochlin bound to heparin but not to several extracellular matrix proteins. Each hCochlin-BWZ cells were cultured in a well coated with type I collagen (col I), type II collagen (col II), type III collagen (col III), type IV collagen (col IV), fibronectin (fib) or heparin (HP) for 18 h at 37°C and β-galactosidase expression was monitored calorimetrically.
(TIF)

S2 Fig Disaccharide composition analysis of HS/Hep. 2-aminobenzoic acid-labeled disaccharides from HP and its desulfated derivatives were used as standards and separated by ion-exchange chromatography on a column of Hitrap DEAE fast flow. The elution profile of mixed standard disaccharides was shown.
(TIF)

S3 Fig Amount of GAGs and expression of sulfotransferases in young and old mice inner ear tissues. (A) Amount of extracted GAGs from 1 mg inner ear tissues from 8-week-old (young, n = 3) and 13-month-old female C57BL/6 mice (old, n = 3) were measured by carbazole method. (B) The mRNAs of sulfotransferases from inner ear tissues of young and old mice were quantified by using real-time PCR. The amount of mRNA was shown as a relative amount when the amount of GAPDH mRNA is 1.0. *N*-sulfotransferase (*ndst*), HS-6-*O*-sulfotransferase 1 and 2 (*hs6st1* and *hs6st2*). In case of PCR for *ndst*, common primers for all of *ndst1*, *ndst2*, *ndst3*, and *ndst4* were used. The data are represented as the mean ± SD of n = 3 mice. * *p*<0.1.
(TIF)

S1 Table Primer sequences used in this study. (PDF)

S1 Raw images (PDF)

S2 Raw images (PDF)

## Abbreviations

Ab: antibody
LCCL: limulus factor C
COCH: coagulation factor C homology gene
GAG: glycosaminoglycans
ECM: extracellular matrix
CS: chondroitin sulfate
DS: dermatan sulfate
HS: heparan sulfate
HP: heparin
