# Supplementary figures and images for: Involvement of cochlin binding to sulfated heparan sulfate/heparin in the pathophysiology of autosomal dominant late-onset hearing loss (DFNA9)
Source: PLoS One. 2022 Jul 28;17(7):e0268485. doi: 10.1371/journal.pone.0268485 (PMC9333281; doi:10.1371/journal.pone.0268485)

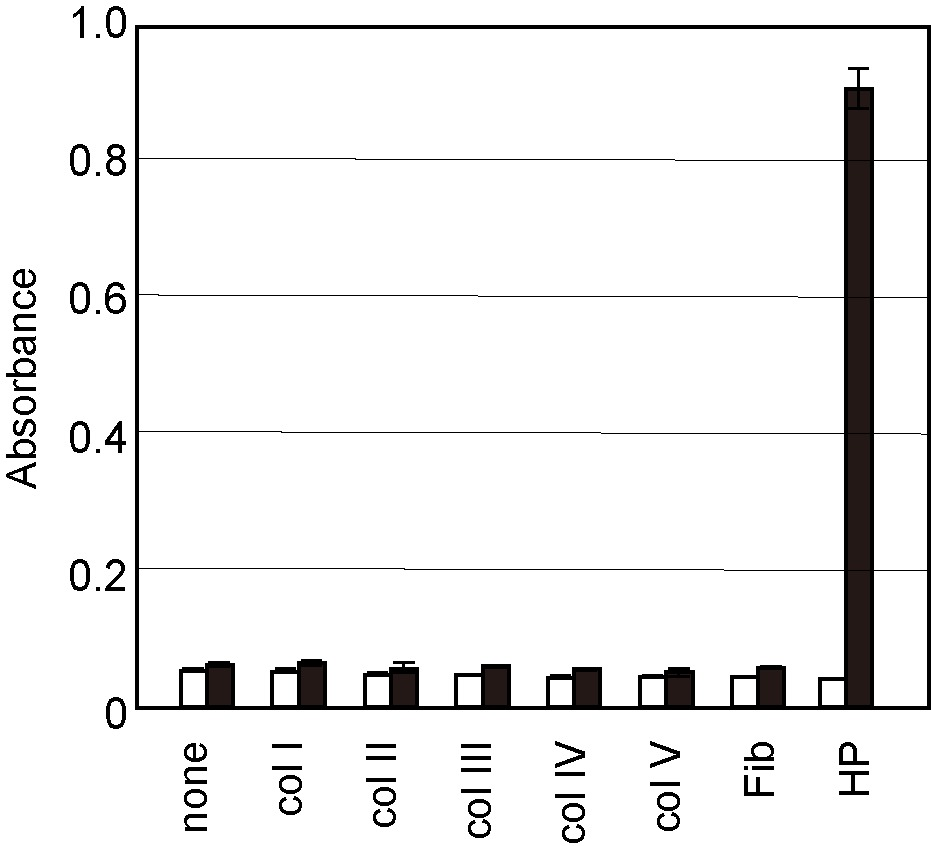

Supplement: S1 Fig — Each hCochlin-BWZ cells were cultured in a well coated with type I collagen (col I), type II collagen (col II), type III collagen (col III), type IV collagen (col IV), fibronectin (fib) or heparin (HP) for 18 h at 37°C and β-galactosidase expression was monitored calorimetrically. (TIF) [file pone.0268485.s001.tif]

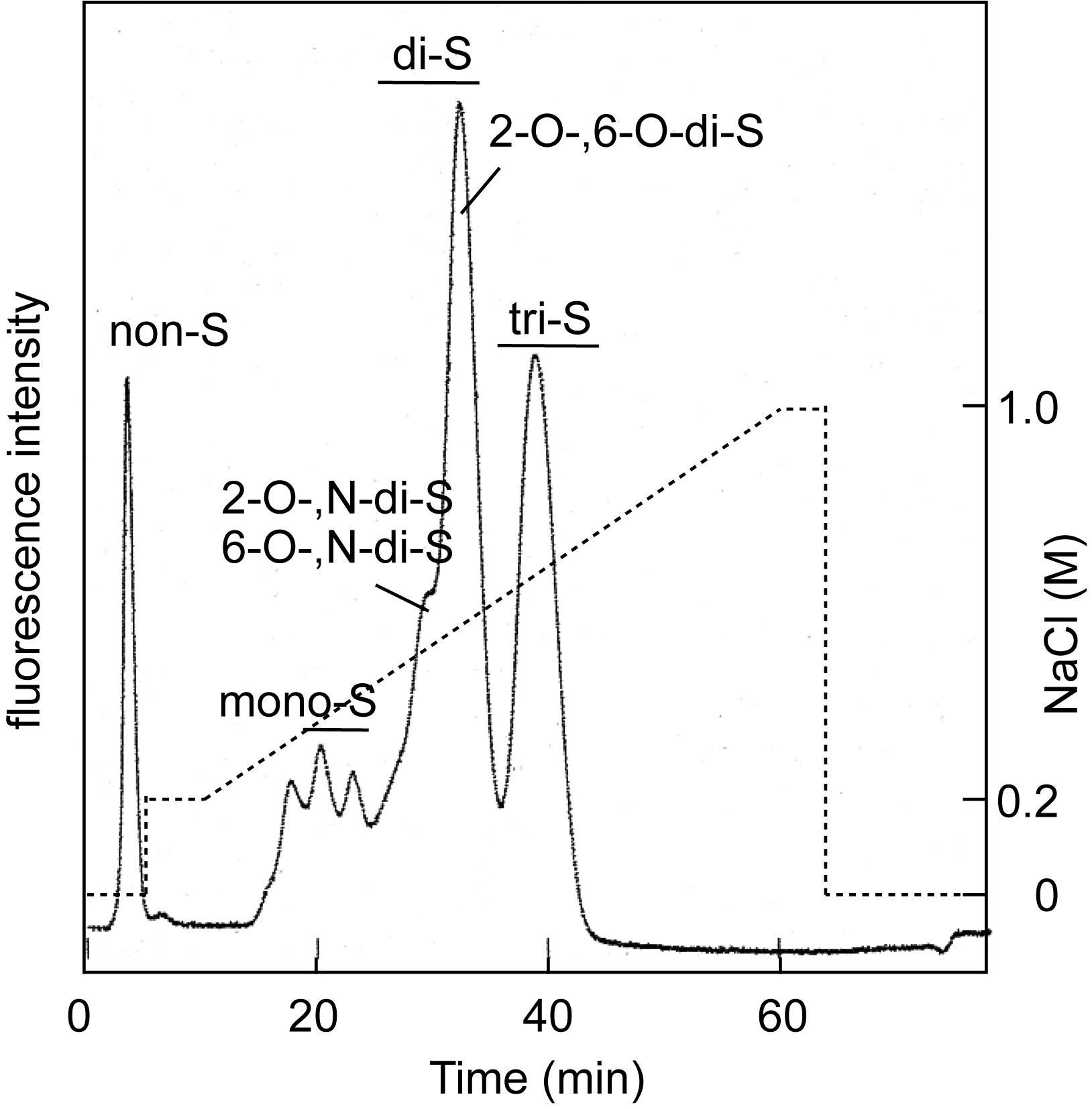

Supplement: S2 Fig — 2-aminobenzoic acid-labeled disaccharides from HP and its desulfated derivatives were used as standards and separated by ion-exchange chromatography on a column of Hitrap DEAE fast flow. The elution profile of mixed standard disaccharides was shown. (TIF) [file pone.0268485.s002.tif]

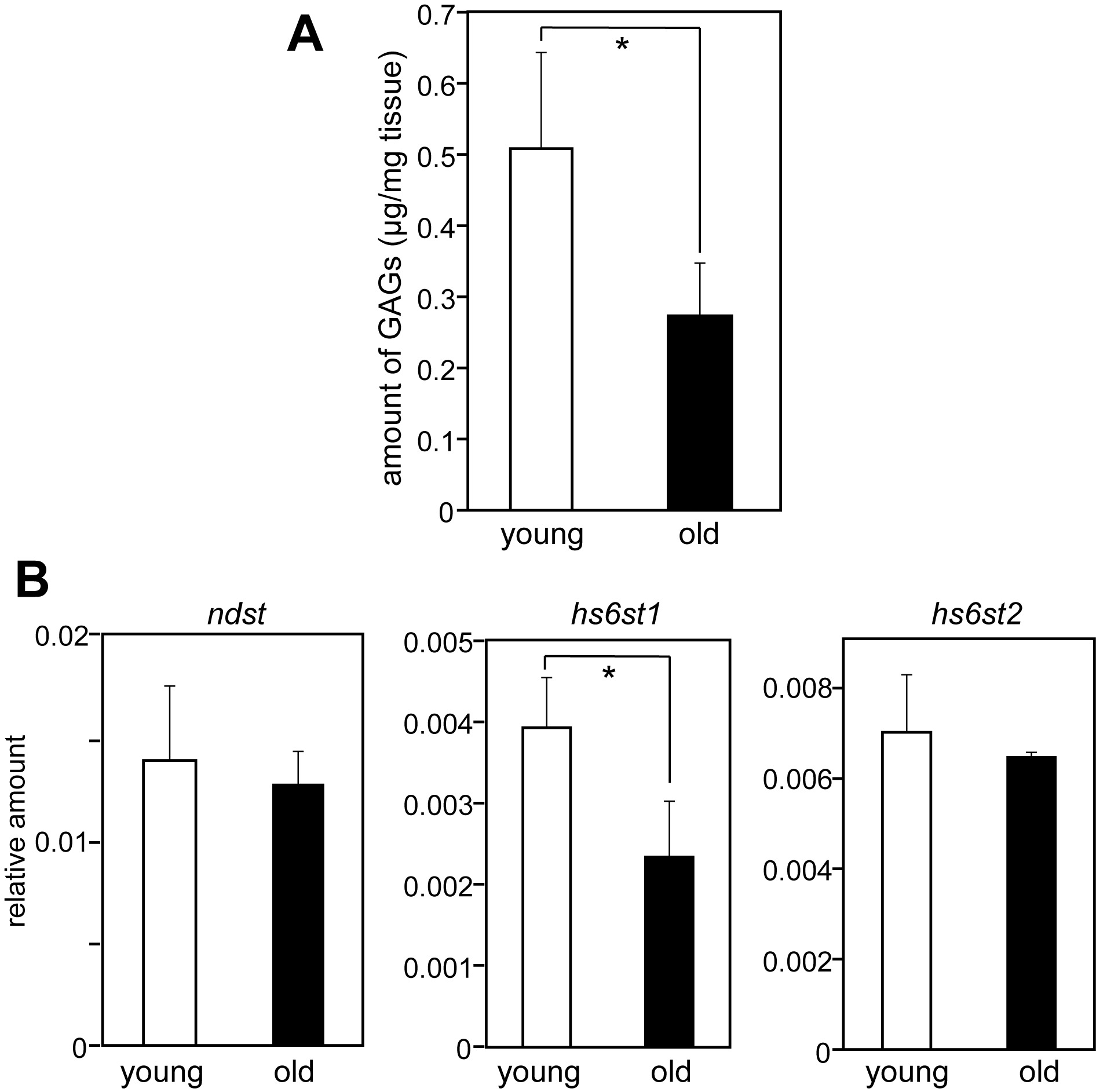

Supplement: S3 Fig — (A) Amount of extracted GAGs from 1 mg inner ear tissues from 8-week-old (young, n = 3) and 13-month-old female C57BL/6 mice (old, n = 3) were measured by carbazole method. (B) The mRNAs of sulfotransferases from inner ear tissues of young and old mice were quantified by using real-time PCR. The amount of mRNA was shown as a relative amount when the amount of GAPDH mRNA is 1.0. N-sulfotransferase (ndst), HS-6-O-sulfotransferase 1 and 2 (hs6st1 and hs6st2). In case of PCR for ndst, common primers for all of ndst1, ndst2, ndst3, and ndst4 were used. The data are represented as the mean ± SD of n = 3 mice. * p<0.1. (TIF) [file pone.0268485.s003.tif]

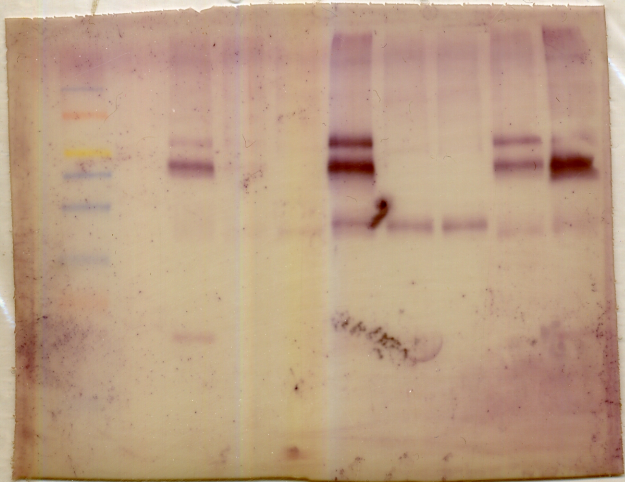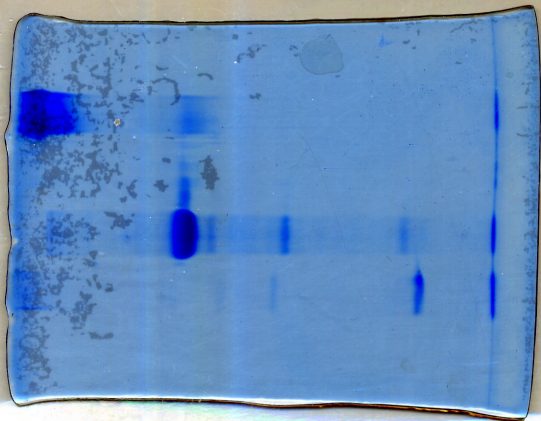

Supplement: S1 Raw images — (PDF) [file pone.0268485.s005.pdf]

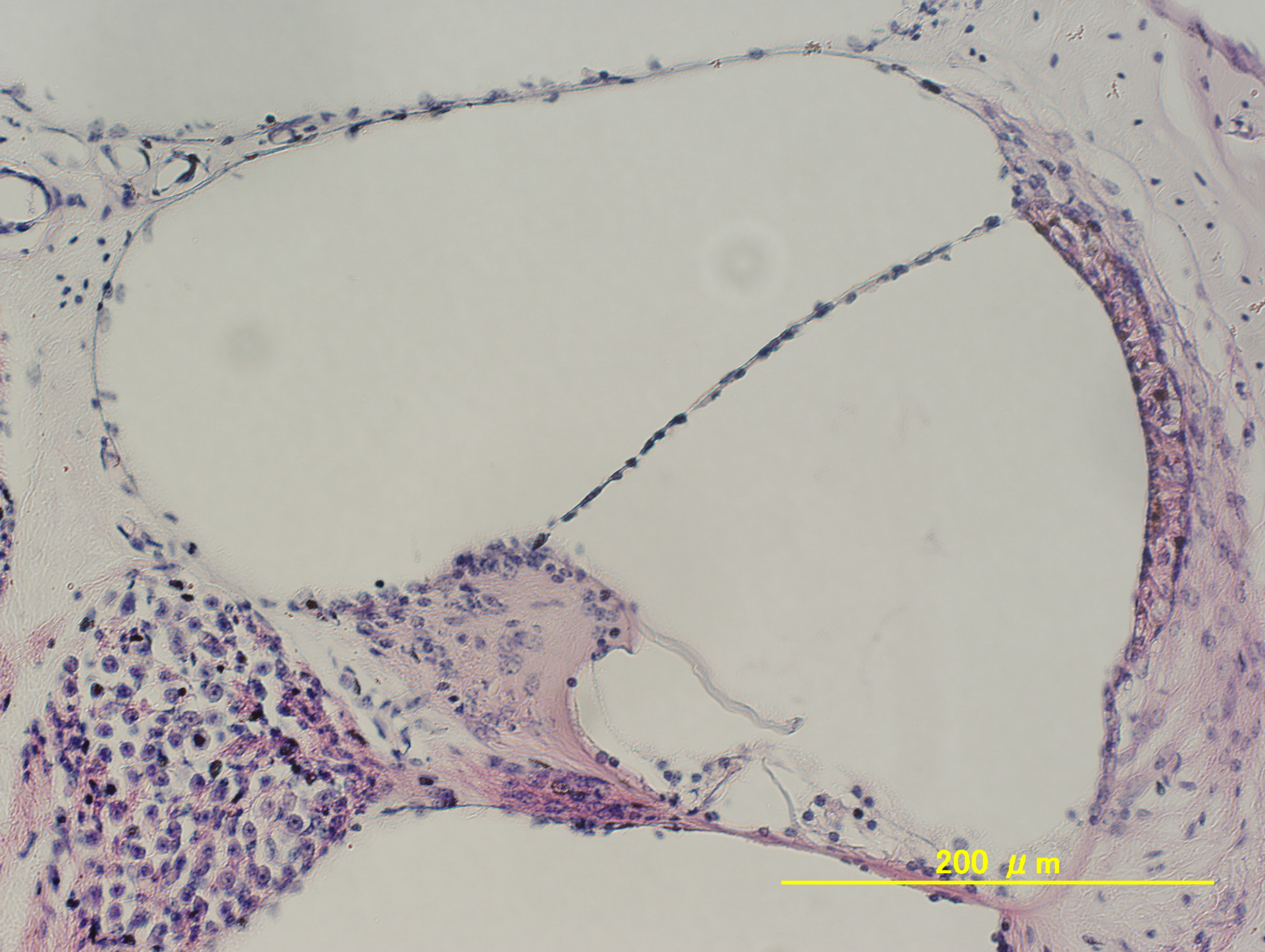

200  $\mu$  m

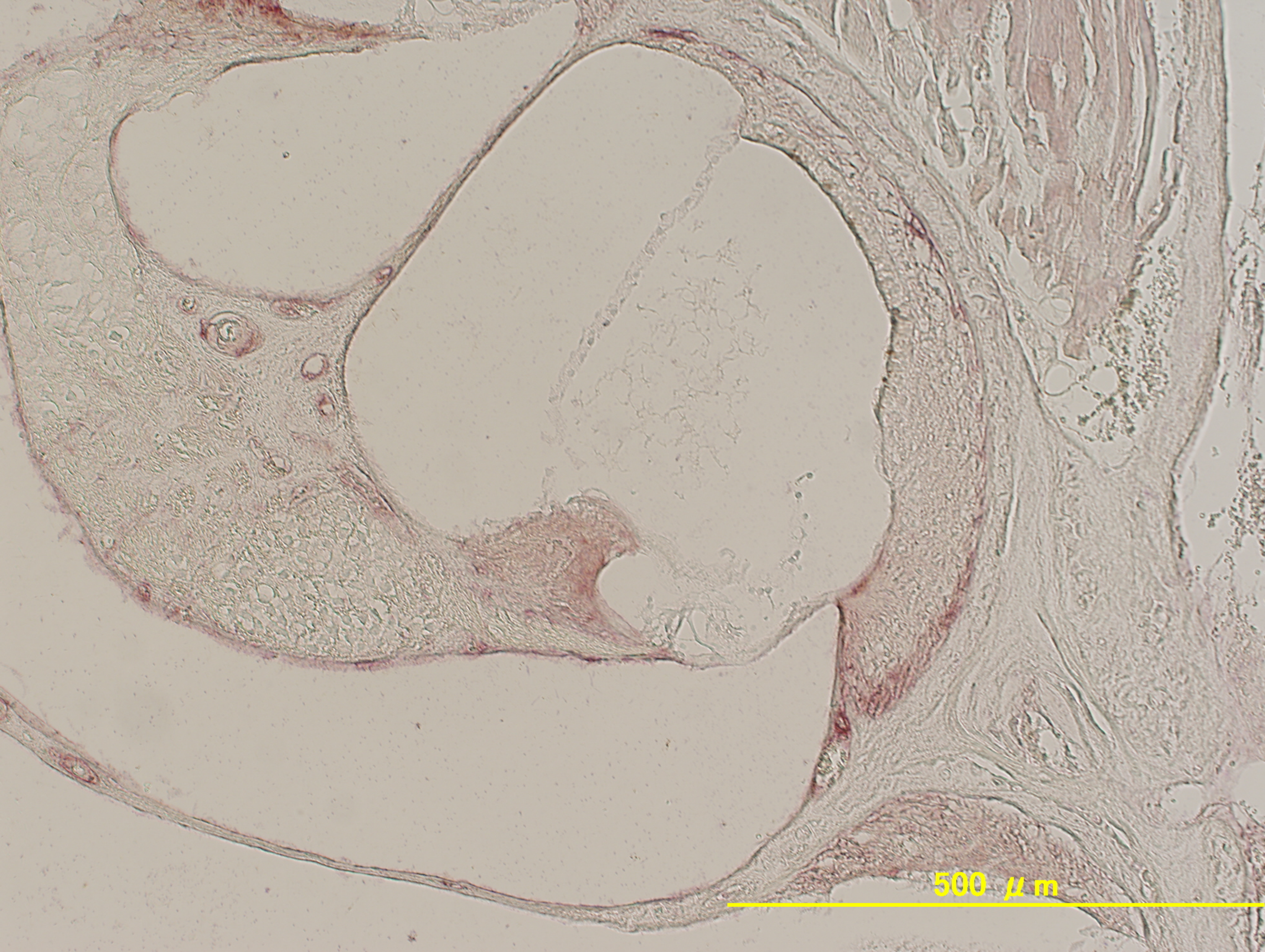

500  $\mu$  m

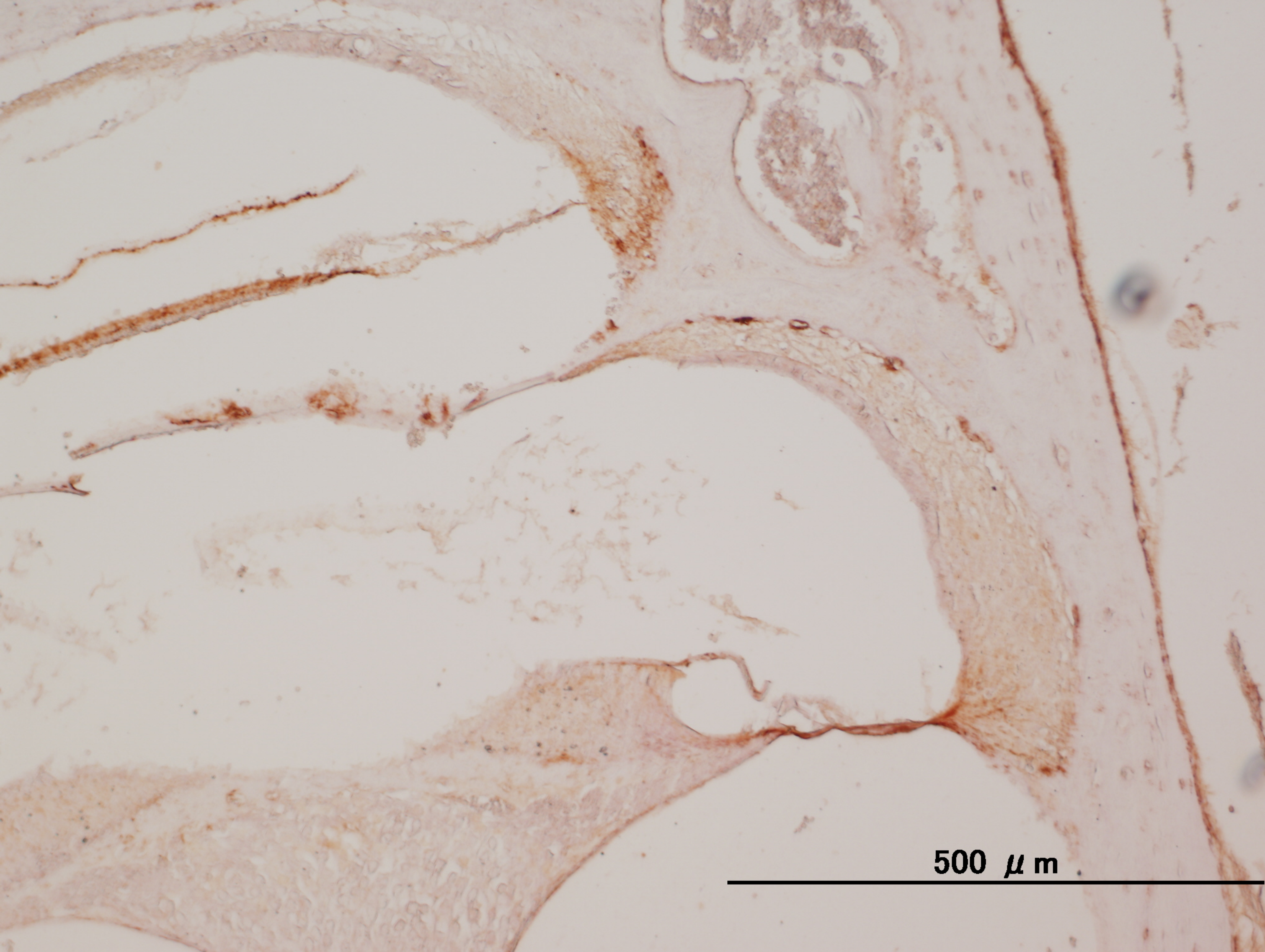

500  $\mu$  m

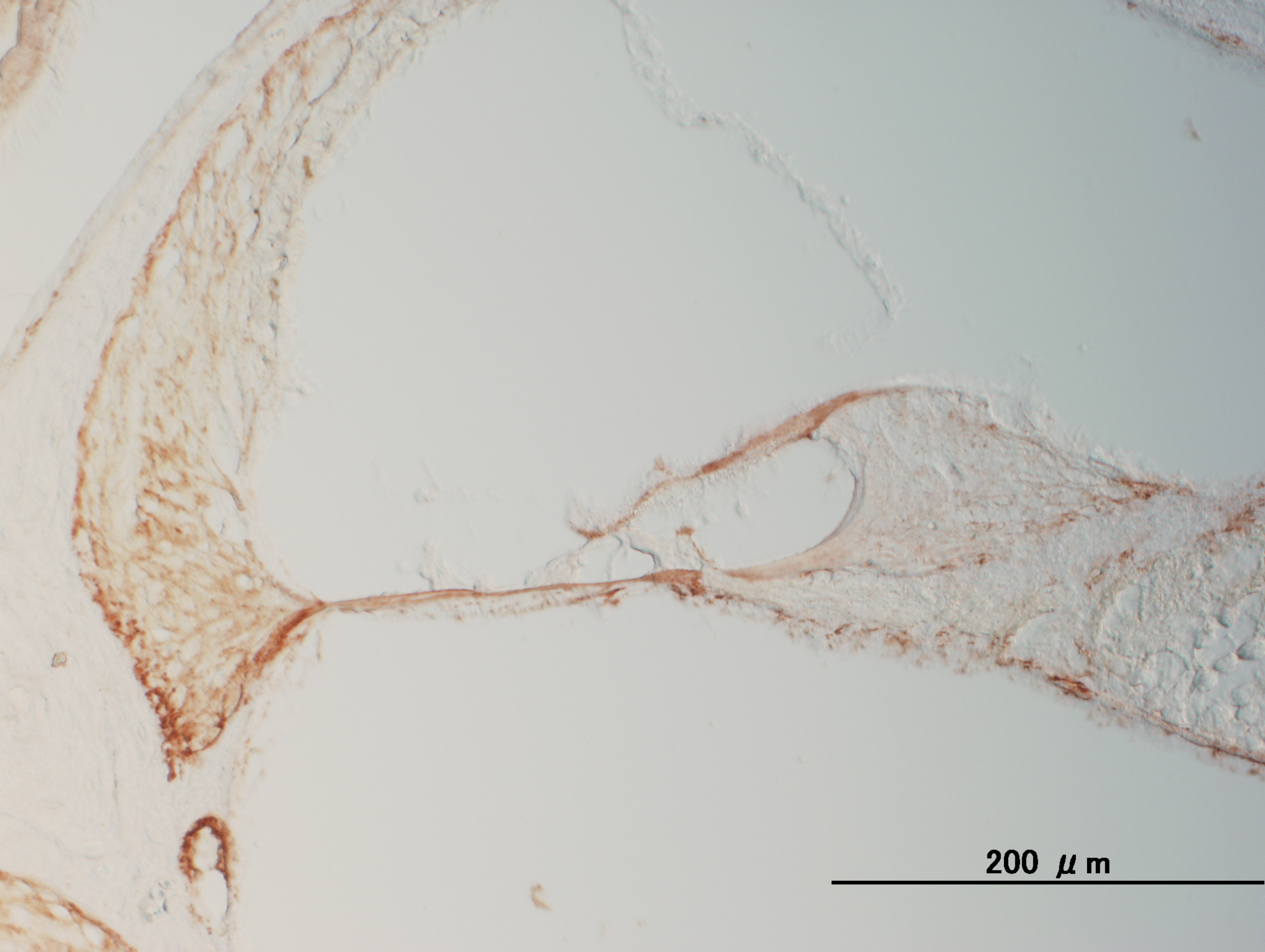

200  $\mu$  m

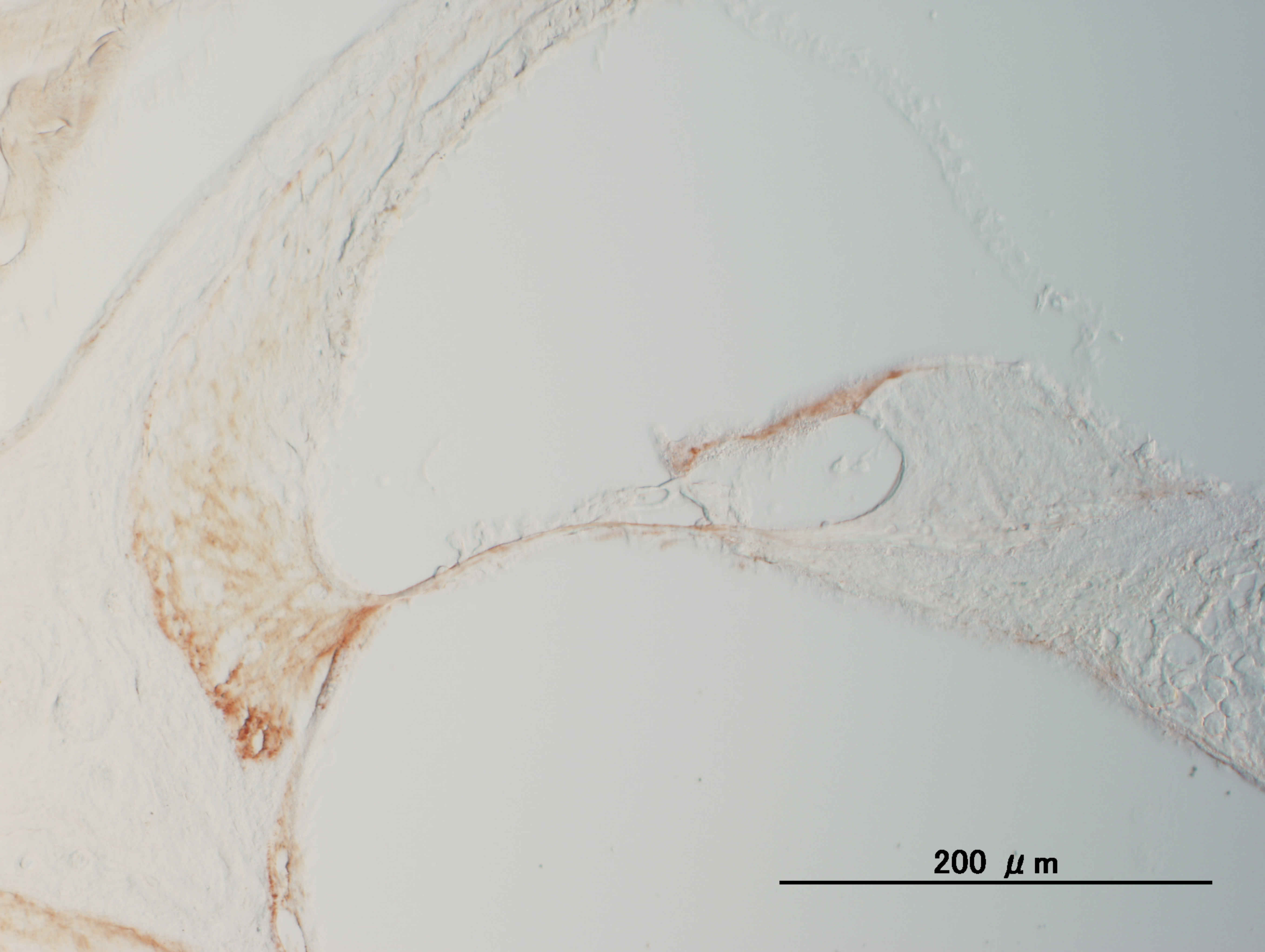

200  $\mu$ m

Supplement: S2 Raw images — (PDF) [file pone.0268485.s006.pdf]
