# Supplementary material for: Involvement of cochlin binding to sulfated heparan sulfate/heparin in the pathophysiology of autosomal dominant late-onset hearing loss (DFNA9)
Source: PLoS One. 2022 Jul 28;17(7):e0268485. doi: 10.1371/journal.pone.0268485 (PMC9333281; doi:10.1371/journal.pone.0268485)
Supplement: S1 Table — (PDF) [file pone.0268485.s004.pdf]

S1 Table 1

mCochlin-F	5'-GTTCTCTGTGTTTGGGAACAT-3'
mCochlin-R	5'-TCCTCAAGAGAGCAGCCTCC-3'
CD3ζ-F	5'-ATCTCGAGTTAAGTTAACAGAGCAAAATTCAGCAGG-3'
CD3ζ-R	5'-TAGCGGCCGCTTAGCGAGGGGCCAGGG-3'
CD8α-F	5'-GTTAACAGTTCTGTCGTGCCAGTC-3'
CD8α-R	5'-TTAGTACTCCTGTGGTAGCAGATGAGAG-3'
CochlinFc-F	5'-ATAAGAATGCGGCCGCGTCACCATGTCCGCAGCC-3'
CochlinFc-R	5'-TTGCTGGGATTCTAAGAAAT-3'
Cochrep-F	5'-TTCTCGAGACCATGTCCGCAGCCT-3'
Cochrep-R	5'-GGCACGACAGAACTGTTAACTTGCTGGGATTCTAAGAAATCTC-3'
FLAG-Cochrep-F	5'-TTGAATTCGGATCCCTCGAGGCCGCTCCCATTGCTATC-3'
FLAG-F	5'-TATTAATTAAACCATGTCTGCACTTCTGATC-3'
FLAG-R	5'-TTCTCGAGGGATCCGAATTCCTTGTCATCGTCATCCTTGTA-3'
Mock-F	5'-TTCTCGAGGTTAACAGTTCTGTCGTGCCAGTC-3'
P51S-sense	5'-GATGTCCTCTGCTCAGGGGGCTG-3'
P51S-antisense	5'-CAGCCCCCTGAGCAGAGGACATC-3'
V66G-sense	5'-GTATGGGAACATAGGATATGCTTCTGTATC-3'
V66G-antisense	5'-GATACAGAAGCATATCCTATGTTCCCATAC-3'
G87W-sense	5'-CAGCAACTCATGGGGACCTGTACG-3'
G87W-antisense	5'-CGTACAGGTCCCCATGAGTTGCTG-3'
G88E-sense	5'-GCAACTCAGGGGAACCTGTACGAG-3'
G88E-antisense	5'-CTCGTACAGGTTCCCCTGAGTTGC-3'
V104Δ-sense	5'-GAAAACTATTCCTCAGATGCCAATGGCATC-3'
V104Δ-antisense	5'-GATGCCATTGGCATCTGAGGAATAGTTTTC-3'
I109N-sense	5'-GATGCCAATGGCAACCAGTCTCAAATG-3'
I109N-antisense	5'-CATTTGAGACTGGTTGCCATTGGCATC-3'
W117R-sense	5'-GCTTTCTAGACGGTCTGCTTCTTTCAC-3'
W117R-antisense	5'-GTGAAAGAAGCAGACCGTCTAGAAAGC-3'
A119T-sense	5'-GCTTTCTAGATGGTCTACTTCTTTCACA-3'
A119T-antisense	5'-GTTACTGTGAAAGAAGTAGACCATCTAGAAAGC-3'
C542F-sense	5'-GTCATCAGAGGCATTTTTAGAGATTTCTTAGAATC-3'
C542-antisense	5'-GATTCTAAGAAATCTCTAAAAATGCCTCTGATGAC-3'
